# Isoniazid and rifapentine treatment effectively reduces persistent *M*. *tuberculosis* infection in macaque lungs

**DOI:** 10.1172/JCI161564

**Published:** 2022-09-15

**Authors:** Riti Sharan, Shashank R. Ganatra, Dhiraj K. Singh, Journey Cole, Taylor W. Foreman, Rajesh Thippeshappa, Charles A. Peloquin, Vinay Shivanna, Olga Gonzalez, Cheryl L. Day, Neel R. Gandhi, Edward J. Dick, Shannan Hall-Ursone, Smriti Mehra, Larry S. Schlesinger, Jyothi Rengarajan, Deepak Kaushal

**Affiliations:** 1 Southwest National Primate Research Center, Texas Biomedical Research Institute, San Antonio, Texas, USA.; 2 National Institute of Allergy and Infectious Diseases, Bethesda, Maryland, USA.; 3 University of Florida College of Pharmacy, Gainesville, Florida, USA.; 4 Emory Tuberculosis Center and; 5 Emory Vaccine Center, Emory National Primate Research Center, Emory University, Atlanta, Georgia, USA.; 6 Department of Medicine, Division of Infectious Diseases, Emory University School of Medicine, Atlanta, Georgia, USA.

**Keywords:** Infectious disease, Tuberculosis

## Abstract

A once-weekly oral dose of isoniazid and rifapentine for 3 months (3HP) is recommended by the CDC for treatment of latent tuberculosis infection (LTBI). The aim of this study is to assess 3HP-mediated clearance of *M. tuberculosis* bacteria in macaques with asymptomatic LTBI. Twelve Indian-origin rhesus macaques were infected with a low dose (~10 CFU) of *M. tuberculosis* CDC1551 via aerosol. Six animals were treated with 3HP and 6 were left untreated. The animals were imaged via PET/CT at frequent intervals. Upon treatment completion, all animals except 1 were coinfected with SIV to assess reactivation of LTBI to active tuberculosis (ATB). Four of 6 treated macaques showed no evidence of persistent bacilli or extrapulmonary spread until the study end point. PET/CT demonstrated the presence of significantly more granulomas in untreated animals relative to the treated group. The untreated animals harbored persistent bacilli and demonstrated tuberculosis (TB) reactivation following SIV coinfection, while none of the treated animals reactivated to ATB. 3HP treatment effectively reduced persistent infection with *M. tuberculosis* and prevented reactivation of TB in latently infected macaques.

## Introduction

Most people infected with *Mycobacterium tuberculosis* do not progress to active tuberculosis (ATB) but instead contain the bacteria and develop asymptomatic, latent tuberculosis (TB) infection (LTBI) (1). However, these individuals remain at risk for developing ATB disease, for example, when coinfected with HIV (2). The commercial tests available to detect LTBI — the tuberculin skin test (TST) (2) and ELISA-based Interferon Gamma Release Assays (IGRAs) (3) — fail to determine whether an individual has cleared infection or harbors persistent bacilli. The CDC recommends the use of a once-weekly regimen of isoniazid and rifapentine for 3 months (3HP) for treatment of LTBI in humans (4). 3HP is effective at reducing the risk of developing ATB (5), suggesting that it mediates clearance of *M*. *tuberculosis* in LTBI. However, the sterilizing efficacy of the regimen on *M*. *tuberculosis* has not been demonstrated. Thus, a better understanding of treatment-mediated clearance of *M*. *tuberculosis* infection is needed in order to improve monitoring and evaluation of treatment regimens for LTBI.

The nonhuman primate (NHP) model is attractive for studying human *M*. *tuberculosis* infection and for performing preclinical studies on treatment regimens, as it recapitulates key aspects of human *M*. *tuberculosis* infection states and TB (6). A majority of rhesus macaques infected with low-dose *M*. *tuberculosis* CDC1551 via aerosolization develop asymptomatic LTBI (7, 8). Moreover, coinfecting latently *M*. *tuberculosis–*infected macaques with SIV results in reproducible reactivation (9). Thus, the NHP model allows us to gain longitudinal and mechanistic insights into the efficacy of treatment regimens for *M*. *tuberculosis*, including in lung compartments, which is difficult to investigate in humans. Between 2014 and 2017, we conducted studies of LTBI and SIV-induced reactivation of tuberculosis (TB) in rhesus macaques to evaluate the efficacy of the 3HP regimen (10). We discovered irregularities in the timing and frequency of treatment in a subset of the animals reported in that study, which led us to subsequently retract the published work (10). Here, we repeated the study to investigate the persistence of *M*. *tuberculosis* in the lungs of asymptomatic rhesus macaques with long-term *M*. *tuberculosis*–infection and to evaluate the efficacy of 3HP in eradicating persistent *M*. *tuberculosis* in a macaque model of 3HP treatment. To assess the effectiveness of 3HP in clearing *M*. *tuberculosis* infection, we coinfected both treated and untreated animals with SIV. Our results clearly suggest that the 3HP treatment is efficacious, leading to substantial reduction in clinical signs of TB, bacterial burden, granuloma numbers, volume of inflammation, and degree of disease.

## Results

### Clinical correlates of LTBI, 3HP treatment, and TB reactivation in rhesus macaques.

**Figure 1:**
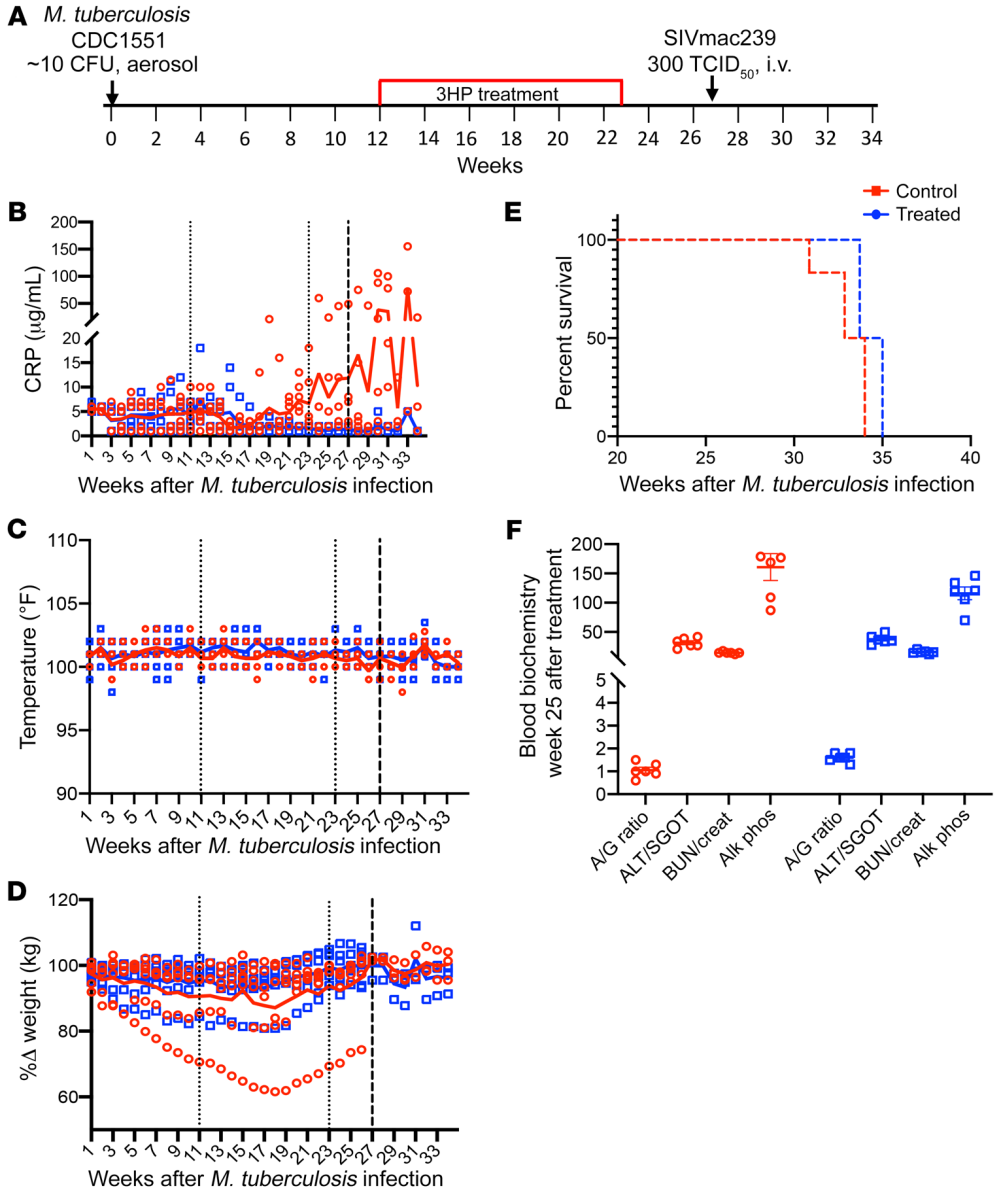
Study outline and clinical parameters. (**A**) The nonhuman primate (NHP) model of latent tuberculosis infection and treatment. Animals were infected with 10 CFU of *M*. *tuberculosis* CDC1551, and of the 12 animals that developed latent tuberculosis infection, 6 were left untreated, while 6 were treated weekly with isoniazid and rifapentine for 3 months (3HP) and rested for 1 month before coinfection with SIV. (30) Animals were monitored for signs of disease such as (**B**) C-reactive protein (CRP), (**C**) pyrexia, and (**D**) wasting throughout the study. The thin dotted lines represent the treatment period and the thick dotted line represents SIV coinfection. The solid lines are the corresponding trendlines for each set of data.(**E**) Survival kinetics shown as days after *M*. *tuberculosis* infection. (**F**) Blood biochemistry for serum albumin/globulin (A/G) (g/dL) ratio, aspartate aminotransferase or serum glutamic-oxaloacetic transaminase (ALT/SGOT) (units per liter of serum), blood urea nitrogen/creatinine (BUN/creat) (μmol/L) ratio, and alkaline phosphatase (Alk phos) (units per liter), at week 25 after TB infection or 1-week after treatment completion for both treated and control groups. The small dotted lines at weeks 12 and 23 mark the 3HP treatment period, while the bold dotted line at week 27 marks the SIV coinfection time point

Twelve animals were exposed to a low dose of *M*. *tuberculosis* CDC1551 (Figure 1A). Infection was confirmed by a positive TST (2) at weeks 3 and 5 after *M*. *tuberculosis* infection. All animals in the study developed LTBI infection, characterized by the absence of culturable bacilli in bronchoalveolar lavage (BAL), serum C-reactive protein (CRP) less than or equal to 10 μg/mL (Figure 1B), and no significant changes in body temperature (Figure 1C) and body weight (Figure 1D) for up to 12 weeks after *M*. *tuberculosis* infection. One group (*n =* 6) remained untreated, whereas the second group (*n =* 6) was treated with the once-weekly 3HP regimen for 12 weeks. One month after treatment completion (i.e., 7 months after *M*. *tuberculosis* infection), coinfection with SIV led to TB reactivation in the majority of untreated animals, as demonstrated by increased CRP levels (Figure 1B). One of the animals in this group (31438) progressed to active TB by week 18 (evident from increased CRP levels in this animal, Figure 1B, and greater than 20% weight loss, Figure 1D). Therefore, this animal was not coinfected with SIV and was instead euthanized 32 weeks after TB infection (its data were included in the data analysis). Due to the clinical signs and symptoms of TB reactivation, which were CRP levels greater than or equal to 10 μg/mL, greater than 20% weight loss, loss of appetite, and increased lesions as seen via PET/CT, the control animals were humanely euthanized (Figure 1E). While 3 animals demonstrated more weight loss compared with the others during the treatment period, the weight loss was not significantly greater nor consistent for more than 2 weeks in the same animal. There was no significant difference between CRP values of untreated and 3HP-treated animals at week 3 (*P* = 0.91), week 9 (*P* = 0.61), or week 23 (0.08) after *M*. *tuberculosis* infection. However, there were significant differences in the CRP levels after SIV coinfection at necropsy between the 2 groups (*P* = 0.01). Importantly, none of the 3HP-treated animals exhibited elevated CRP levels (Figure 1B), pyrexia (Figure 1C), or wasting (Figure 1D) after SIV coinfection and did not need to be euthanized due to disease progression (Figure 1E). These animals were subsequently euthanized for necropsy and tissue collection at week 34 after TB infection. No significant differences were observed in blood biochemistry (Figure 1F) between the 2 groups following 3HP treatment completion, confirming absence of drug-induced cytotoxicity. These results indicate that a majority of macaques in this study were infected with *M*. *tuberculosis* for >28 weeks and remained asymptomatic until substantial immune perturbation occurs via SIV coinfection. However, it is possible that a percentage of macaques could have reactivated had they been left untreated.

### PET/CT imaging analysis of TB reactivation.

**Figure 2:**
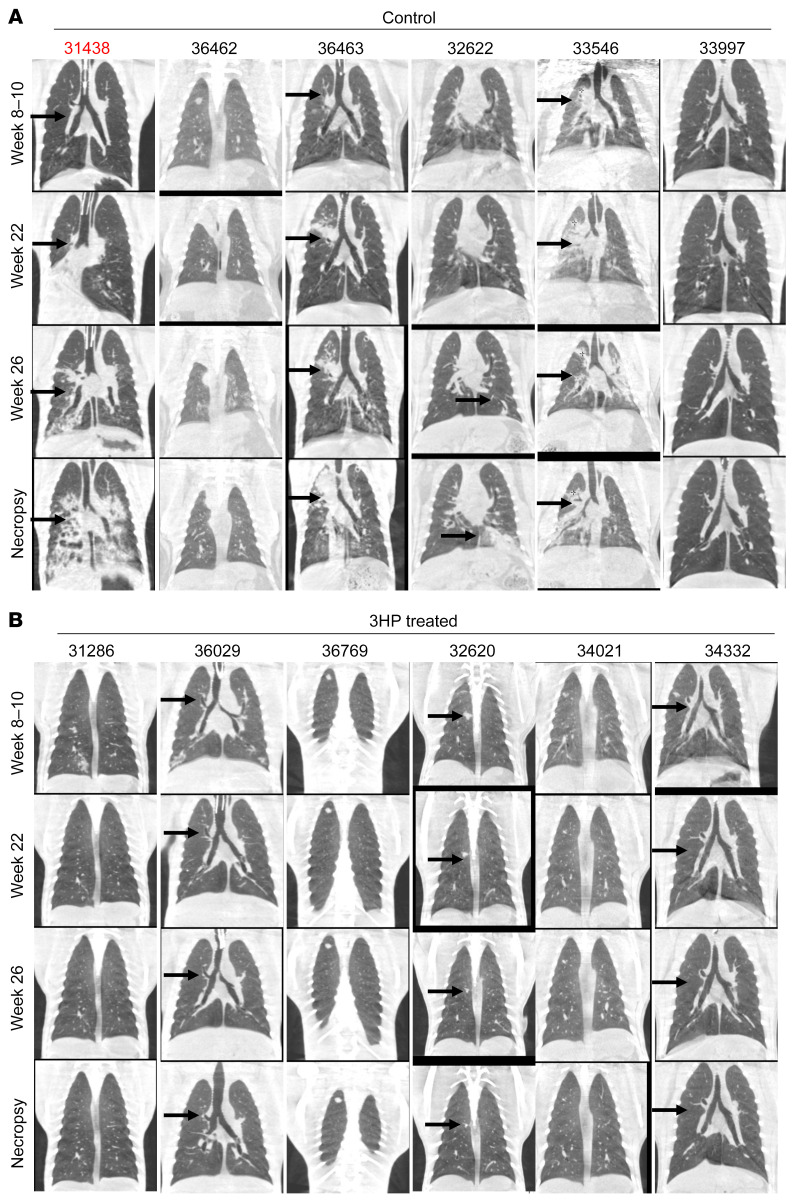
CT imaging of treated versus control macaques. CT scans of (**A**) control and (**B**) 3HP-treated rhesus macaques at weeks 8–10, 22, and 26 after TB infection and at study end point. Animal 31438 was an active progressor and was not administered SIV. In the longitudinal CT scans performed, macaques in the 3HP treatment group reported resolving lung lesions as early as 2–4 weeks after 3HP treatment initiation (black arrows), while there were no new lung lesions, and preexisting lung lesions resolved further at 10 weeks after 3HP initiation (black arrows).

Coinfection with SIV led to TB reactivation in untreated animals, as demonstrated by the presence of numerous granulomatous lesions detected by CT scans (Figure 2A). While a solitary macaque (33997) exhibited spontaneous reactivation prior to SIV coinfection and exhibited many lesions, the other 5 untreated, SIV coinfected animals had clear evidence of granulomatous lesions as well. Animal 36462 had comparatively less evidence of progression. Furthermore, the 3HP-treated animals did not demonstrate the presence of increased lesion numbers after SIV coinfection (Figure 2B, marked with black arrow). The lung lesions in all macaques remained stable, i.e., no or minimal progression in size and architecture at weeks 8–10 after infection, confirming LTBI (Figure 2, A and B, marked with black arrow). Five of 6 macaques in the control group showed gradual progression in TB pathology after SIV coinfection, with multiple new lung lesions and increased size of already established nodular lung lesions (Figure 2A, marked with black arrow).

**Figure 3:**
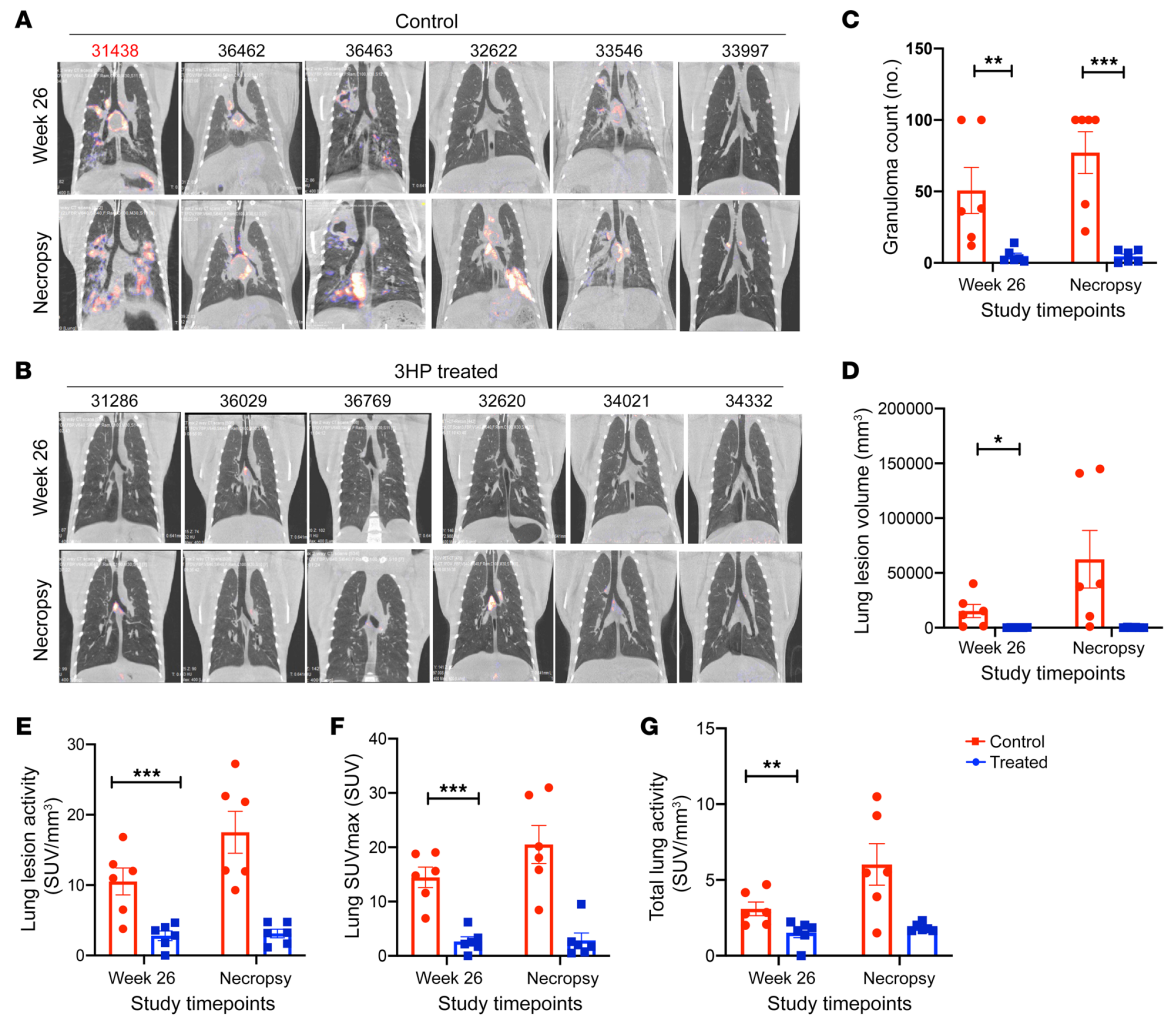
PET scans of treated and control rhesus macaques. (**A**) PET scans of 6 untreated control animals demonstrating gradual progression in TB pathology from week 26 after TB infection up to necropsy with multiple new lung lesions, and increased size of previously reported nodular lung lesions. (**B**) PET scans of 6 animals treated with 3HP demonstrating no new lung lesions, (**C**) granuloma counts, (**D**) lung lesion volume, (**E**) lung lesion activity, (**F**) lung SUVmax, and (**G**) total lung activity at weeks 26 and necropsy in treated and control animals. Data are represented as mean ± SEM. Significance was determined using 2-way ANOVA or multiple 2-tailed *t* tests using Holm-Šidák method, *P < 0.05; **P <0.01; ***P < 0.001.

TB pathogenesis and the efficacy of the 3HP prophylaxis regimen were examined using PET/CT scans (11) (Figure 3). All of the macaques in the study had focal nodular lung opacities, while 9 of the 12 displayed mild-to-moderate lymph node enlargement by 5–6 weeks after aerosol *M*. *tuberculosis* infection. The 18F-fluorodeoxyglucose (18F-FDG) scans were performed 3 weeks after completion of 3HP regimen, i.e., week 26, in all animals (Figure 3, A and B). These scans clearly revealed both the presence of persistent foci of increased FDG uptake in the controls (Figure 3A), and the effectiveness of the 3HP regimen (Figure 3B) at the completion of the treatment. After SIV infection, scans in the treated group reported few to no new lung lesions, while the already established lung lesions did not increase in size, and no increase in FDG uptake (Figure 3B) was observed in the majority of the animals in this cohort. In contrast, 5 of6 control (untreated) animals showed an increase in size of lung lesions and increased FDG standard uptake values (SUV) (Figure 3A), signifying reactivation and further progression of lung TB pathology. All 6 untreated control animals showed involvement of multiple lung lobes, with some examples of consolidation, lobar collapse, cavitary lesions, and massive mediastinal lymph node enlargement, after SIV coinfection. The number (*P* = 0.0181) (Figure 3C) and volume of lung lesions (*P* = 0.0335) (Figure 3D), lung lesion activity (*P* = 0.0002) (Figure 3E), SUVmax (*P* = 0.0036) (Figure 3F), and total lung activity (*P* = 0.0335) (Figure 3G) of control animals were each significantly higher compared with the 3HP treatment group after treatment completion. Our results, therefore, suggest effective resolution of lung TB lesions after prophylactic treatment with the 3HP regimen.

### 3HP treatment–mediated clearance of persistent M. tuberculosis infection in macaques.

**Figure 4:**
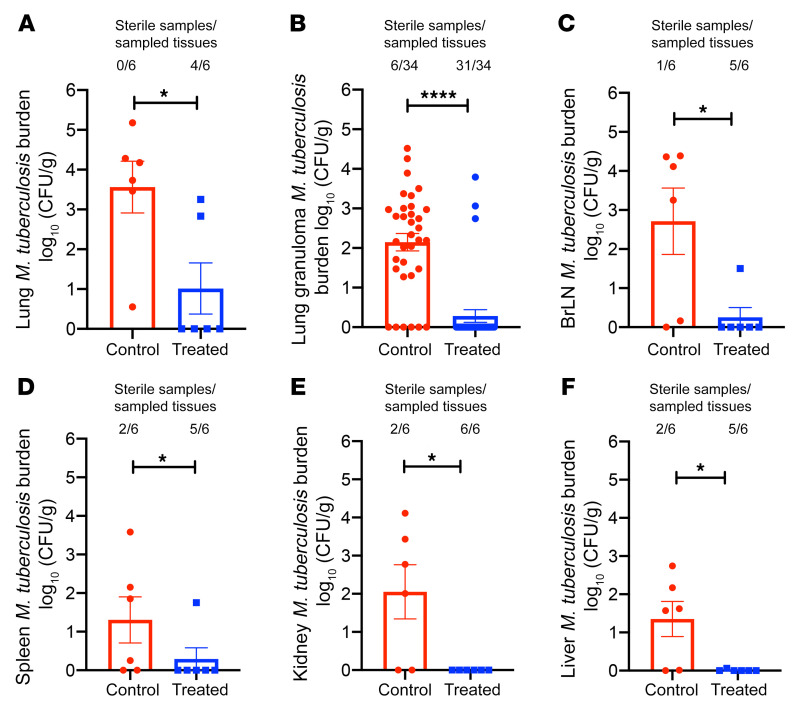
Bacterial persistence and burden. (**A**) Lung bacterial burden in animals that were left untreated for 7 months compared with animals treated with 3HP, which mirrored results found in (**B**) lung granulomas. Dissemination and extra thoracic bacterial burden were further measured in (**C**) bronchial lymph nodes (BrLN), (**D**) spleen, (**E**) kidney, and (**F**) liver. *P < 0.05, and ****P < 0.0001 using Student’s 2-tailed *t* test.

To assess *M*. *tuberculosis* bacterial burdens in pulmonary and extrapulmonary compartments of 3HP-treated and untreated animals following SIV coinfection, lungs and other organs were assayed for *M*. *tuberculosis* by culture at necropsy (Figure 4). The lung bacterial CFU loads in the untreated group (mean of 3.56 log_10_) were significantly higher than in the 3HP-treated group (mean of 1.0 log_10_; *P* = 0.0085) (Figure 4A). All 6 of the untreated animals harbored bacilli in their lungs, while 4 of the 6 3HP-treated animals were completely devoid of any replicative bacilli, despite 50% of the lung tissue being used for CFU analyses. In addition to assessing the bacterial burden in random sections, we also identified and isolated individual granulomas from the 2 groups of animals. We observed significantly higher bacterial burdens in the granulomas of untreated animals (*P* < 0.0001) compared with 3HP-treated animals (Figure 4B). In the treated group, only 3 of 34 individual granulomas (8%) harbored culturable bacilli compared with the untreated group, where 32 of 34 granulomas (94%) harbored replicative bacilli (Figure 4B). Statistically significantly higher bacterial burdens were also observed in extrapulmonary organs: bronchial lymph nodes (*P* = 0.02) (Figure 4C), spleen (*P* = 0.01) (Figure 4D), kidney (*P* = 0.01) (Figure 4E), and liver (*P* = 0.01) (Figure 4F). Only 1 of6 treated animals exhibited culturable *M*. *tuberculosis* in bronchial lymph nodes and spleen, and none of the animals harbored bacteria in the liver or kidney.

### Pulmonary pathology in 3HP-treated and untreated macaques.

**Figure 5:**
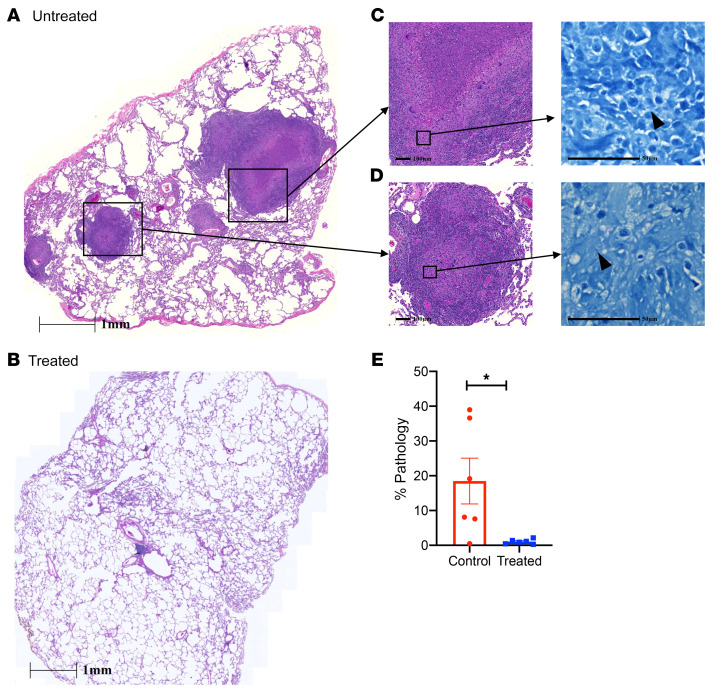
Pulmonary pathology. Lung tissue at the time of necropsy was stereoscopically distributed for analysis by H&E staining. (**A** and **B**) Histologic analysis of lung tissues at study end point after SIV coinfection in (**A**) untreated animals and (**B**) treated animals. Scale bars: 1 mm. (**C** and **D**) A representative image demonstrates severe pathology and bacterial burden in multiple areas such as (**C**) bronchial lumen and (**D**) lymphangitic lesions, with indicated scale bars for each image. Arrowheads denote acid-fast bacilli present after Ziehl-Nielsen staining. Scale bars: 100 μm (left), 50 μm (right). (**E**) Analysis of animals treated with 3HP demonstrated significantly lower to no detectable granuloma lesions or severe consolidation prominent in coinfected animals, as shown by histologic analysis. *P < 0.05 using Student’s 2-tailed *t* test. % Pathology, percentage of lung involvement in each group.

To determine the effect of 3HP treatment on lung pathology, lung tissue was collected at necropsy and subjected to H&E staining to study the cellular and granulomatous pathology (Figure 5 and Supplemental Figure 1; supplemental material available online with this article; https://doi.org/10.1172/JCI161564DS1). The pathological findings were correlated with the clinical and microbiological findings. All 6 untreated control animals demonstrated granulomas in lung tissue at necropsy (Figure 5A), whereas 4 of the 6 3HP-treated macaques demonstrated no granulomas (Figure 5B) in the lung tissue. Detailed histopathological analysis of stereologically collected samples from all animals demonstrated robust granulomatous inflammation in the untreated group, suggestive of SIV-induced reactivation. Untreated animals demonstrated well-formed granulomas with caseous central areas (Figure 5C) and multifocal histiocytic to mixed inflammation (immature granulomas) (Figure 5D). Digital quantification of lung pathology showed significantly higher (*P* = 0.02) lung involvement (mean of 18%; range, 7%–39%) in the untreated control group compared with the 3HP-treated group (mean of 1%; range, 0.28%–2.15%) (Figure 5E). Disseminated granulomatous inflammation — in the bronchial lymph nodes, spleen, and liver — was observed in 4 of6 animals in the untreated group and in 1 of6 animals in the treated group (data not shown).

### Immunologic and virologic effects of SIV infection in LTBI macaques.

**Figure 6:**
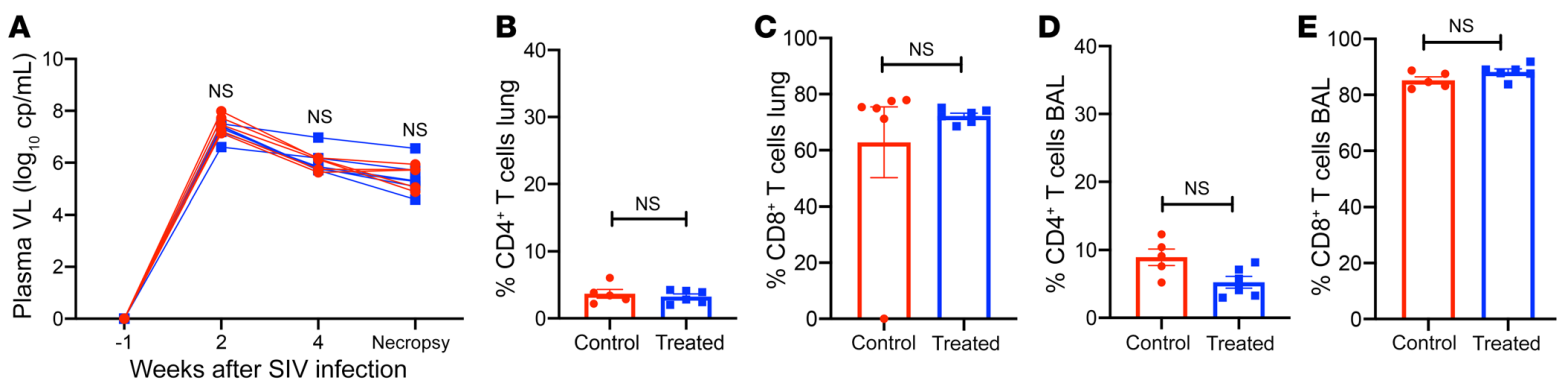
Immune measurements. (**A**) Plasma viral loads (Plasma VL) after SIV infection demonstrate parallel viral infection and burden in both groups. (**B**–**E**) Analysis of CD4^+^ and CD8^+^ T cells as a percentage of CD3^+^ lymphocytes by flow cytometric analysis of single-cell suspensions in (**B** and **C**) lung cells and in (**D** and **E**) BAL at the time of necropsy. No significance was found using (**A**) 2-way ANOVA with Šidák’s correction or (**B**–**E**) Student’s 2-tailed *t* test.

SIV plasma viral loads were measured in each animal to rule out the possibility that the differences in the clinical outcomes between treated and untreated groups were due to differential viral replication (Figure 6A). No statistically significant differences were observed in the viral loads at both the acute set point and end stage of SIV infection between the 2 groups (Figure 6A). Flow cytometric analysis of BAL and lung cells from 3HP-treated and untreated animals that were obtained at necropsy following SIV coinfection showed that the frequencies of CD4^+^ T cells in the lungs of both groups of animals were comparable (7%–9%; no statistical difference) (Figure 6, B and C). Lung CD8^+^ T cells were equally elevated in both groups (>75%) and were statistically indistinguishable (Figure 6, B and C). Similarly, in BAL, there was a comparable depletion of CD4^+^ T cells in both groups, while the frequencies of CD8^+^ T cells were elevated (Figure 6, D and E).

## Discussion

Our study demonstrates that viable *M*. *tuberculosis* can persist within the lungs of rhesus macaques for up to 7 months during the asymptomatic LTBI state. Furthermore, we were able to assess the effectiveness of 3HP treatment for clearing *M*. *tuberculosis* in a model of LTBI and SIV-mediated reactivation. Our data show that the 3HP regimen was able to clear *M*. *tuberculosis* in 4 of6 treated macaque lungs and prevent reactivation of LTBI in all 6 treated animals following SIV coinfection. In comparison, all 6 untreated animals demonstrated clear signs of TB reactivation upon SIV coinfection. The CDC recommends 3HP as an effective treatment for LTBI in humans, and our study shows low levels of culturable bacteria in the lungs of 3HP-treated NHPs. Our study does not establish complete sterilization of *M*. *tuberculosis* bacilli by 3HP, as treated animals may harbor low numbers of bacteria that are unable to cause disease within the study period. Overall, our study establishes what we believe to be a new animal model for evaluating the efficacy of drug regimens such as 3HP, which can be extended to study additional treatment regimens for LTBI. Moreover, this model allows for detailed immunologic and microbiological investigations in local and peripheral compartments during persistent *M*. *tuberculosis* infection, treatment, and reactivation to TB disease.

*M. tuberculosis* is able to reside within the lung tissue in a slow or nonreplicating state due to its resistance to host immunity and ability to withstand hypoxia and oxidative stress (12). Although LTBI is associated with low-level persistence of *M*. *tuberculosis* without progression to disease, current diagnostics cannot detect *M*. *tuberculosis* in asymptomatic IGRA-positive individuals. As a result, we are unable to identify the subset of IGRA-positive and/or TST-positive individuals harboring viable bacilli in their lungs versus those who may have cleared infection. Furthermore, studying lung-specific host immune responses associated with LTBI in humans remains challenging (13). Our model allows for longitudinal sampling over long periods of time to monitor clinical, radiologic, pathologic, microbiologic, and immunologic parameters subsequent to precise delivery of *M*. *tuberculosis* via aerosol. Thus, this model provides a platform for further, more detailed investigation into immune correlates of persistence or clearance of *M*. *tuberculosis* infection. Analogous to IGRA-positive patients who do not develop TB disease, we found that a majority of macaques in our study remained devoid of clinical signs of TB following a low-dose infection with *M*. *tuberculosis* CDC1551 (7). Moreover, a substantial reactivation to TB following SIV coinfection confirmed the presence of viable *M*. *tuberculosis* bacilli in these animals. The early progression to ATB before SIV coinfection in a minority of macaques represents a caveat of our model. This is likely due to the fact that, while we exposed animals to low doses of *M*. *tuberculosis*, exposures of 10–20 CFU are still probably significantly higher than the physiological exposure of most humans. Early progression to TB in our model may be analogous to individuals who progress to primary TB relatively early after infection. We believe that while the limitations of our model do not diminish the overall significance of our findings, it is nevertheless important to recognize these issues when applying this model to future studies. Characterization of LTBI in cynomolgus macaques by a heterogeneous mixture of sterile and nonsterile granulomas has also been reported (11). It is believed that local physiology, oxygenation status, and local lung immune responses play a critical role in the balance between control of persistent bacilli during LTBI and active replication of *M*. *tuberculosis* during progression to TB (14). Thus, our animal model provides the advantage of studying lung immune responses longitudinally, which is difficult to study in humans (15, 16).

The CDC currently recommends the 3HP regimen as preventive treatment for LTBI in the United States and notes that a shorter 3HP regimen leads to substantially higher completion rates,compared with a 9-month regimen of isoniazid alone (17). However, the metrics for evaluating the success or failure of any treatment regimen center on epidemiologic rates of TB relapse or recurrence (18). Using our rhesus macaque model of LTBI, we were able to directly assess 3HP-mediated clearance of persistent *M*. *tuberculosis* bacilli. We show that 3HP treatment markedly reduced (persistent *M*. *tuberculosis* burdens, as shown by PET/CT scans, microbiological culture, and lack of LTBI reactivation upon SIV coinfection. 3HP-mediated *M*. *tuberculosis* clearance was also independent of differences in SIV viral loads or depletion of CD4^+^ T cells in BAL and lung, which were comparable in treated and untreated animals. These results suggest that the extent of pathology observed in these untreated animals resulted from recent reactivation of TB infection following SIV coinfection rather than progression of disease from the *M*. *tuberculosis* infection 8–9 months earlier. Drug hepatotoxicity, leading to lower rates of patient adherence is often seen during LTBI treatment. Scale up of LTBI treatment globally would be significantly affected by reductions in the treatment duration (19). In addition to being cost-effective and causing less hepatotoxicity, the shortened duration and frequency of 3HP dosing has resulted in much higher rates of treatment completion (20, 21). Similar to humans, we observed no hepatotoxicity in the study animals after completion of 3HP treatment. The comparative clinical trials between once-weekly 3HP and daily isoniazid alone for 9 months examined the percentage of patients that developed TB after treatment as the main end point (4, 5). Additionally, a 1-month regimen of daily isoniazid-rifapentine (1HP) in patients with HIV infections living in areas of high tuberculosis prevalence was noninferior to 9 months of isoniazid alone in preventing tuberculosis in this cohort (22). Our macaque model demonstrates effective clearance of *M*. *tuberculosis* infection by the 3HP regimen and provides evidence that 3HP reduces persistent *M*. *tuberculosis* infection.

One of the limitations of our study is the inability to precisely model latently infected people who remain asymptomatic for extended periods of time after their initial exposure to *M*. *tuberculosis*. Rather, our model of LTBI and its treatment more closely models recently infected contacts of TB-source cases with positive IGRA and TST, but who fail to progress to ATB within the first year after exposure. Given that the highest risk of developing TB is in the first 2 years after exposure, recent contacts are considered to be a priority for preventive treatment (23). Another limitation is the use of a single agent, SIV, to induce LTBI reactivation in our model. Future studies can test additional agents such as tumor necrosis factor blockade or steroid-mediated immunosuppression to induce LTBI reactivation. We were also limited by the inability to assess the effect of 3HP on nonculturable bacilli. While 3HP effectively clears culturable *M*. *tuberculosis*, we are unable to currently determine its effect on nonculturable bacilli.

### Conclusions.

Through our NHP model of TB, we have demonstrated that *M*. *tuberculosis* can persist in the lungs of latently infected macaques for months after infection, effectively modeling IGRA-positive contacts of TB cases in humans with LTBI. Furthermore, we provide experimental evidence of the 3HP regimen as preventive treatment for LTBI by showing that treatment with 3HP reduced the risk of developing TB in our macaque-LTBI model. Together, these results confirm clinical studies on 3HP and we believe that they establish a robust preclinical NHP platform for immunologic investigations of LTBI and evaluation of novel drug candidates and regimens for treating contacts of drug-sensitive and drug-resistant TB cases.

## Methods

### Animal infection and 3HP treatment.

12 naive, Indian-origin rhesus macaques were infected via aerosol with a low dose (~10 CFU) of *M*. *tuberculosis* CDC1551 (Supplemental Table 1; refs 7, 8, 16). Infection was confirmed by TSTs at weeks 3 and 5 after infection. Animals were monitored for CRP, body weight, and body temperature weekly. All animals were TST positive, but remained devoid of ATB for up to 12 weeks, and thus were considered to have developed LTBI. They were randomly assigned to either treatment or control groups (6 animals each). The treatment group received a weekly oral dose of 15mg/kg isoniazid and 15 mg/kg rifapentine for 12 weeks, which began at week 12 after aerosol infection and lasted to week 23 after TB infection. Oral intake was monitored by veterinary staff to ensure consumption. To confirm clearance of *M*. *tuberculosis* bacilli by 3HP treatment, 11 of12 animals were coinfected with a 300 median–tissue culture infectious dose of SIV_mac239_ intravenously at week 27 after *M*. *tuberculosis* infection (7, 9). Animals were euthanized upon signs of ATB — such as a strong PET/CT signal, presence of culturable *M*. *tuberculosis* in BAL, continuous weight loss, and high serum CRP levels and anorexia — or as time-matched controls.

### PET/CT imaging.

Longitudinal CT and PET/CT scans were performed using a LFER150 PET/CT scanner (Mediso) at 3- to 6-week intervals, starting from week 4 after TB infection with the last scan prior to necropsy (24). Briefly, we performed 18F-FDG PET/CT scans for each anesthetized macaque using the breath-hold technique (25). All of the animals received an intravenous injection of 5 mCi dose of 18F-FDG (26), procured from Cardinal Health radiopharmacy. The single field-of-view (FOV) and/or double FOV lung CT scans were performed using breath-hold as described (27). PET scans were acquired after completion of the 40–50 minute FDG–uptake period. Images were visualized using Interview Fusion 3.03 (Mediso) and reconstructed using Nucline NanoScan LFER 1.07 (Mediso), with parameters as described (28). The lung segmentation, volumetric, and SUV analyses were performed using Vivoquant 4.0 (Invicro) (24).

Briefly, the region of interest (ROI) (29) was drawn using connected thresholding referencing Hounsfield units for the lung, while also drawn manually, to identify lung lesions as previously described (30). Subsequently, image-derived mean SUVs were calculated for the complete lung ROI and represented as total lung activity, while the mean SUV for the lung lesion ROI are represented as lung lesion activity (31). The SUVmax of the 18F-FDG in the lungs of the TB-infected macaques, usually seen in the lung lesions, is represented as lung SUVmax. Granuloma count was performed by identifying and counting heterogenous TB lesions manually (32). Animals showing greater than 100 granulomas and/or consolidation or collapse of granulomas are represented as TNTC, or too numerous to count.

### Assessment of M. tuberculosis infection and disease.

Weekly physical examinations including measurement of body weight, temperature, and SIV viral load were determined as previously described (7, 16, 33, 34). Bacterial burden associated with *M*. *tuberculosis* infection was determined at necropsy by plating homogenized tissue sections, as described previously (7, 8, 16). Individual lung lobes were sectioned into 5 μm thick samples and stereologically selected for analysis that allowed for unbiased selection of lung tissue (35). Randomly selected sections were pooled for CFU and used for histopathology. Approximately 50% of the lung tissue was pooled by lung lobe (*n =* 5/animal), homogenized, serially diluted, and plated in triplicate. Approximately 30% of the lung tissue was fixed and stained with hematoxylin and eosin using standard methods for histologic analysis and scanned with Zeiss axio scan.Z1 slide scanner at ×40 magnification and the digital slides were analyzed using an optimized tissue classifier in HALO v3.3 software (Indica Labs). The remaining tissue was processed as single-cell suspensions for flow cytometry as described previously (7, 16). Bronchial lymph nodes, spleen, liver and kidney were plated for CFUs. All infected macaques were housed in Animal Biosafety Level 3 facilities (ABSL3 facilities) at the Southwest National Primate Research Center (SNPRC), where they were treated according the standards recommended by the Association for Assessment and Accreditation for Lab Animal Care International (AAALAC International) and the NIH guide for the Care and Use of Laboratory Animals.

### Statistics.

Statistical analysis was performed using GraphPad Prism (version 8.4.1). A *P* value of less than 0.05 was considered statistically significant. Data are represented as mean ± SEM. Specific analysis are indicated in the figure legend of each figure and include 2-tailed Student’s t tests or 2-way ANOVA with Holm-Šidák multiple comparison test as applicable.

### Study approval.

The study procedures were approved by the Animal Care and Use Committee of the Texas Biomedical Research Institute. Animals were anesthetized and intubated under the supervision of a board-certified veterinarian as per approved Texas Biomedical Research Institute IACUC protocols.

## Author contributions

DK and JR conceptualized and funded this research. DK, RS, JR, SM, TWF, and CLD designed the experiments. RS, SRG, DKS, JC, RT, CAP, VS, OG, EJD, SHU, and SM performed the experiments. RS, SRG, TWF, RT, CAP, VS, OG, EJD, SHU, SM, DK, and JR analyzed the results. RS, DK, NG, LSS, and JR wrote the manuscript.

## Supplementary Material

Supplemental data

## Version 1 07/21/2022

In-Press Preview

## Version 2 09/15/2022

Electronic publication
