# Supplementary material for: Isoniazid and rifapentine treatment effectively reduces persistent M. tuberculosis infection in macaque lungs
Source: J Clin Invest. 2022 Sep 15;132(18):e161564. doi: 10.1172/JCI161564 (PMC9479578; doi:10.1172/JCI161564)
Supplement: Supplemental data [file jci-132-161564-s078.pdf]

Animal ID	Group	Sex	Age (Years)	3HP Initiation	MAMU Type*		
					*A*01*	*B*08*	*B*17*
32620	3HP treated	Male	8	12 weeks post-TB	Negative	Negative	Positive
34021	3HP treated	Male	6	12 weeks post-TB	Negative	Negative	Negative
34332	3HP treated	Male	7	12 weeks post-TB	Negative	Negative	Positive
31286	3HP treated	Female	10	12 weeks post-TB	Negative	Negative	Positive
36029	3HP treated	Male	5	12 weeks post-TB	Negative	Negative	Positive
36769	3HP treated	Male	4	12 weeks post-TB	Negative	Negative	Negative
32622	Untreated	Male	8	No treatment	Negative	Negative	Negative
33546	Untreated	Female	6	No treatment	Negative	Negative	Negative
33997	Untreated	Male	7	No treatment	Negative	Positive	Negative
31438	Untreated	Female	10	No treatment	Neagtive	Negative	Positive
36462	Untreated	Female	10	No treatment	Negative	Negative	Negative
36463	Untreated	Female	10	No treatment	Negative	Negative	Negative

*MAMU Type: The major histocompatibility complex is key to initiating adaptive immune responses in vertebrates. Orthologues of the HLA class I and II genes have been indentified in rhesus macaques named as *Mamu-A, Mamu-B* . Shown here are the MHC genotyping of the study macaques.

Table 1.

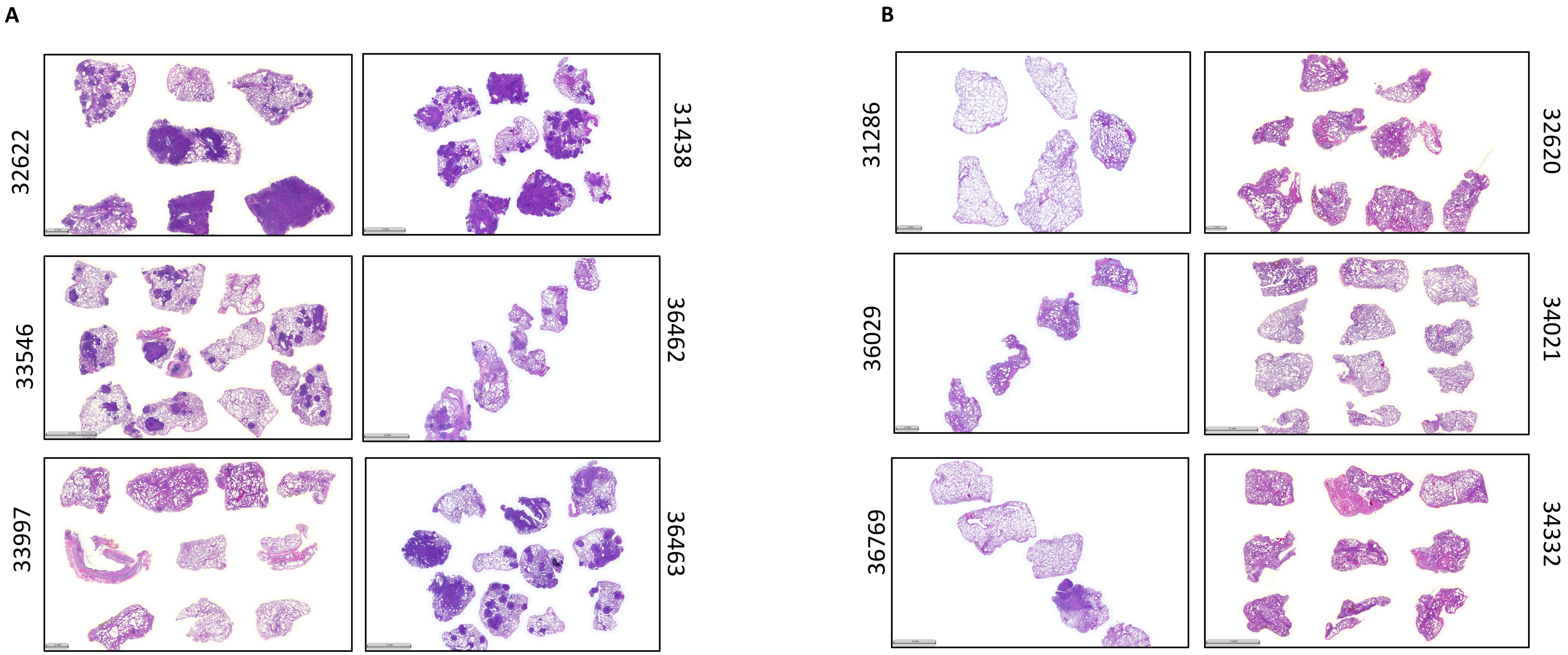
A
B
32622
31438
33546
36462
33997
36463
31286
32620
36029
34021
36769
34332

Figure S1.

Figure S1. Gross pathology of lung tissue at study end point in (A) untreated control and (B) 3HP-treated rhesus macaques. Detailed histopathology analysis of stereologically collected samples from all animals demonstrated robust granulomatous inflammation in the untreated group, suggestive of SIV-induced reactivation. Untreated animals demonstrated well-formed granulomas with caseous central areas, multifocal histiocytic to mixed inflammation (immature granulomas) and increased bronchus-associated lymphoid tissue.
